# Visual detection of Coxsackievirus A6 using a reverse transcription polymerase spiral reaction method

**DOI:** 10.3389/fcimb.2025.1563495

**Published:** 2025-05-13

**Authors:** Kun Wang, Yuanhang Ai, Juan Luo, Longying Liang, Weiwei Zhang, Guojun Cao, He Zha, Jie Wu, Kun Lei, Shifei Yao, Kaifeng Wu

**Affiliations:** ^1^ Department of Laboratory Medicine, The Third Affiliated Hospital of Zunyi Medical University (The First People’s Hospital of Zunyi), Guizhou, China; ^2^ Department of Pediatrics and Child Health, The Third Affiliated Hospital of Zunyi Medical University (The First People’s Hospital of Zunyi), Guizhou, China; ^3^ Department of Clinical Laboratory, Fudan University Huashan Affiliated Hospital, Shanghai, China; ^4^ Scientific Research Center, The Third Affiliated Hospital of Zunyi Medical University (The First People’s Hospital of Zunyi), Guizhou, China

**Keywords:** enteroviruses, Coxsackievirus A6, reverse transcription polymerase spiral reaction, visual detection, hand-foot-mouth disease

## Abstract

Coxsackievirus A6 (CVA6) ranks as a primary enterovirus associated with hand-foot-mouth disease (HFMD) and herpangina (HA). Given its significant role in these diseases, there is an urgent need for an efficient identification method. This study presents a novel visual approach based on the reverse transcription polymerase spiral reaction (RT-PSR) for the rapid detection of CVA6. We designed an RT-PSR assay that targets and amplifies a segment of the VP1 gene. Hydroxy naphthol blue (HNB) is incorporated as the detection agent in this assay. To evaluate the performance of the RT-PSR assay, we analyzed 142 clinical throat swab samples. The results were benchmarked against those obtained using quantitative reverse transcription - polymerase chain reaction (qRT - PCR). The RT-PSR assay operates at 65°C for 60 minutes and exhibits a detection limit of 10 copies/μL. When tested against other viruses, it consistently yielded negative results, demonstrating its high specificity. Moreover, the RT - PSR assay showed excellent agreement with a commercial qRT - PCR kit. In conclusion, by using HNB as an indicator, the RT - PSR assay emerges as a straightforward and highly sensitive method for detecting CVA6 in symptomatic throat samples. This approach holds great potential for improving the diagnosis and surveillance of CVA6 - related diseases.

## 1 Introduction

Hand, foot, and mouth disease (HFMD), caused by enteroviruses, is a global public health issue, especially affecting infants and children. Once dominated by Coxsackievirus A16 (CVA16) and Enterovirus 71 (EV71), recent years have seen a surge in CVA6 cases, triggering pandemics in multiple regions (Zhang et al., 2021; Lim et al., 2016; Gopalkrishna and Ganorkar, 2021; Kamau et al., 2021; Ai et al., 2021). CVA6 is also associated with HFMD in adults (Ramirez-Fort et al., 2014). Currently, there are no specific drugs or vaccines for CVA6, and diagnosis mainly relies on enterovirus nucleic acid testing, crucial for CVA6 - related atypical HFMD (Second et al., 2017).

The gold standard for enterovirus detection, combining virus isolation and serological testing, has drawbacks (Bentley et al., 2021). Virus isolation is complex and time - consuming, and serological screening may yield false negatives, particularly in early infection (Zhou et al., 2022). Nucleic acid amplification tests are sensitive, but conventional methods need sophisticated instruments or complex procedures (Grammatikos et al., 2023). Existing RT - PCR - based enterovirus test kits mainly target EV71 and CVA16, with few of them covering CVA6 (Liu et al., 2014; Zhu et al., 2023). As the epidemiology shifts, there’s an urgent need for a simple, sensitive, and affordable CVA6 diagnostic method, especially in resource - poor areas.

Isothermal nucleic acid amplification like reverse transcription-polymerase spiral reaction (RT-PSR) occurs at a fixed temperature, avoiding costly devices (Huang et al., 2020; He et al., 2020). In comparison with reverse transcription loop-mediated isothermal amplification (RT - LAMP), RT - PSR demands fewer primers, thereby reducing costs (Yadav et al., 2023). The VP1 protein of CVA6, key for host cell infection, is variable and a prime target for molecular typing (Lu et al., 2024a). Hydroxy naphthol blue (HNB), a metal ion indicator, can show amplification in PCR through color change with magnesium ions (Thoraneenitiyan et al., 2022).​

In this study, we developed a rapid CVA6 detection method using RT - PSR, optimized reaction conditions, evaluated performance, and validated it with clinical samples, comparing results with qRT - PCR.

## 2 Materials and methods

### 2.1 Statement on Ethics and Biosafety

The study received ethical approval from the Ethics Committee of the Third Affiliated Hospital of Zunyi Medical University (The First People’s Hospital of Zunyi City; approval number: 2022-202). All experiments were conducted in our biosafety level 2 clinical laboratory.

### 2.2 Virus strains and RNA extraction

The CVA6 viral nucleic acids utilized in this study were isolated and preserved within our laboratory, and their authenticity was confirmed through VP1 sequencing. Other viruses employed as non-target samples in this study, including respiratory syncytial virus, hepatitis C virus, hepatitis B virus, rotavirus, cytomegalovirus, CVA16, EV71 and CVA10 underwent clinically standardized tests. Viral RNA was extracted using the RNA Kit for virus detection (Ref# DP315-R; Tiangen, China). The concentrations of RNA were determined utilizing the NanoDrop™ Lite Spectrophotometer (Thermo Scientific, USA), and subsequently stored at -80°C freezer (Aucma, China) prior to utilization.

### 2.3 The design of primer sets and the construction of a plasmid containing a partial VP1 sequence

The VP1 sequences of CVA6 which were retrieved from the NCBI GenBank database, together with the sequences of CVA6 strains isolated by our group in Zunyi City, were analyzed using the multiple sequence alignment tool Geneious Prime to identify the conserved region of the gene, which served as a template for primer selection. Primers for RT-PSR assay were designed using Oligo 7 software. Three pairs of forward and reverse primers were selected, and an unrelated sequence from a plant source was added to obtain an RT-PSR primer (**Table 1**). To determine the detection limit, a 270 bp fragment containing the target sequence within VP1 coding region was cloned into pGEM-3Zf vectors to generate recombinant plasmids carrying CVA6 (**Table 1**).

**Table 1:** Oligonucleotides used in the study.

Primer name	Sequence (5’ – 3’)	GC%	Positions at VP1*
Primer 1	PSR-F1 acgattcgtacatagaagtatagAACCTCAATGACAGCACGAC	50	403-422
	PSR-R1 gatatgaagatacatgcttagcaAAACCACTGATAAGCCGTTG	45	578-597
Primer 2	PSR-F2 acgattcgtacatagaagtatagGCCCCTAAGCCAGATAGCA	57.89	463-481
	PSR-R2 gatatgaagatacatgcttagcaGGACACTGCCCATATTGCAAA	47.62	642-662
Primer 3	PSR-F3 acgattcgtacatagaagtatagCGCAAGCTAGAGTTGTCAACA	47.62	346-366
	PSR-R3 gatatgaagatacatgcttagcaACTCAATTTTGCGAATACCGAT	36.36	516-537
Primer 4	PSR-F4 gtcaaagcgatcccgccttacAACCTCAATGACAGCACGAC	50	403-422
	PSR-R4 cattccgccctagcgaaactgAAACCACTGATAAGCCGTTG	45	578-597
Primer 5	PSR-F5 gtcaaagcgatcccgccttacGCCCCTAAGCCAGATAGCA	57.89	463-481
	PSR-R5 cattccgccctagcgaaactgGGACACTGCCCATATTGCAAA	47.62	642-662
Primer 6	PSR-F6 gtcaaagcgatcccgccttacCGCAAGCTAGAGTTGTCAACA	47.62	346-366
	PSR-R6 cattccgccctagcgaaactgACTCAATTTTGCGAATACCGAT	36.36	516-537

*Primer location was based on Coxsackievirus A6 92/QJ/YN/CHN/2020/CV-A6 VP1 gene sequence, GenBank entry number: >LC626222.1. The lowercase primer sequence is derived from plants, while the uppercase primer sequence is designed based on the conserved sequence of CVA6 VP1.

### 2.4 Optimization of RT-PSR for the detection of CVA6

The CVA6 RNA, verified by sequencing, was utilized for the optimization of the RT-PSR assay. To determine the occurrence of RT-PSR with these primers, an initial test was conducted on all 6 primers to select those that are suitable. The reaction was conducted in a 25 µl volume comprising of 5 µl RNA templates, 2.5 µl isothermal amplification buffer (10 ×, Ref#M0537S; New England BioLabs, United States), 8.0 mM MgSO_4_ (Ref# M0537S; New England BioLabs, United States), 0.8 M betaine (Ref#B0300; Sigma, United States), 1.4 mM dNTP each (Ref#4019; Takara, Japan), 8 U BcaBEST ^®^ DNA Polymerase ver.2.0 (Ref#RR380A; Takara, Japan), and 7.5 U WarmStart RTx reverse transcriptase (Ref#M0380S; New England BioLabs, United States) along with PSR-F and PSR-R at a concentration of 2.0 µM. The reaction conditions were maintained at a constant temperature of 63°C for 60 minutes. The resulting reaction products were separated through electrophoresis utilizing 1.5% agarose gels.

After determining the primers, the subsequent step involved optimizing the conditions for RT-PSR to detect CVA6. Firstly, the primer concentration was optimized by considering concentrations of 1.6 µmol/L, 2.0 µmol/L, 2.4 µmol/L, 2.8 µmol/L, 3.2 µmol/L and 3.6 µmol/L, respectively. DEPC-treated water was utilized as the negative control (NC). To optimize the efficiency of RT-PSR amplification, we conducted the reaction at various temperatures ranging from 59 to 68°C and determined the optimal assay time by testing four different time points (30 min, 40 min, 50 min, and 60 min). The confirmation was obtained through agarose gel electrophoresis using GoldenView staining (Biomed, China) and visualized with a Gel Doc XR^+^ system (Bio-Rad, United States). As a metal ion indicator, HNB exhibits color transformation in response to the concentration of Mg^2+^ in the reaction solution.

In order to facilitate visual differentiation between negative and positive results, the optimal concentration of Mg^2+^ was determined. The color changes were observed by introducing 2 µL of 1.5 mmol/L HNB (Ref#YB335-H; Yubo Biotechnology, China) indicator into the detection system containing varying concentrations of Mg^2+^ (ranging from 2 mmol/L to 12 mmol/L). A change in color from violet to sky blue indicated a positive result.

### 2.5 Sensitivity test for RT-PSR assay

To assess the sensitivity of the one-step RT-PSR, we determined the copy number of the standard plasmid using the following formula: (copies/µL = 6.02 × 10^23^ × DNA concentration, g/µL)/plasmid length × 660. The plasmid was diluted tenfold with TE buffer to obtain concentrations of (1 × 10^8^, 1 × 10^7^, 1 × 10^6^, 1 × 10^5^, 1 × 10^4^, 1 × 10^3^, 1 × 10^2^, and 1 × 10^1^ copies/µL). Subsequently, we added a volume of five microliters from each dilution to our reaction system. As reverse transcriptase was not required for this sensitivity detection experiment since the plasmid served as our template, DEPC-treated water was used as a negative control. We assessed the sensitivity of the RT-PSR by monitoring changes in color within reaction products and verified the results through subsequent analysis via electrophoresis on 1.5% agarose gels.

### 2.6 Specificity test for RT-PSR assay

The specificity of the RT-PSR assay was evaluated by testing against respiratory syncytial virus, hepatitis C virus, hepatitis B virus, rotavirus, cytomegalovirus, CVA16, EV71 and CVA10. DEPC-treated water served as the negative control during the experiment.

### 2.7 Comparison between RT-PSR and TaqMan Real-Time RT-PCR in clinical samples

To assess the clinical applicability of the RT-PSR method, a consecutive series of 142 clinical throat swab samples were selected for examination. Each throat swab was immersed in 1.5 mL of normal saline and subsequently subjected to percussion blending. A supernatant volume of 200 µL was collected and added to the nucleic acid extraction kit (Ref#RT-B-200; Zybio, China). Viral RNA was extracted from the supernatant using an automated nucleic acid extractor following manufacturer’s instructions. Subsequently, both TaqMan Real-Time RT-PCR (Da AN Gene Co., Ltd, China) and RT-PSR were performed on the extracted RNA. The results obtained from these two methods were compared.

### 2.8 Statistical analysis

Statistical analyses were conducted using in SPSS 18.0 (IBM Corporation, New York, United States). Categorical variables were presented as frequencies and percentages. Kappa statistic was employed to assess the agreement between TaqMan Real-Time RT-PCR and one-step RT-PSR.

## 3 Results

### 3.1 Primers

**Figure 1:**
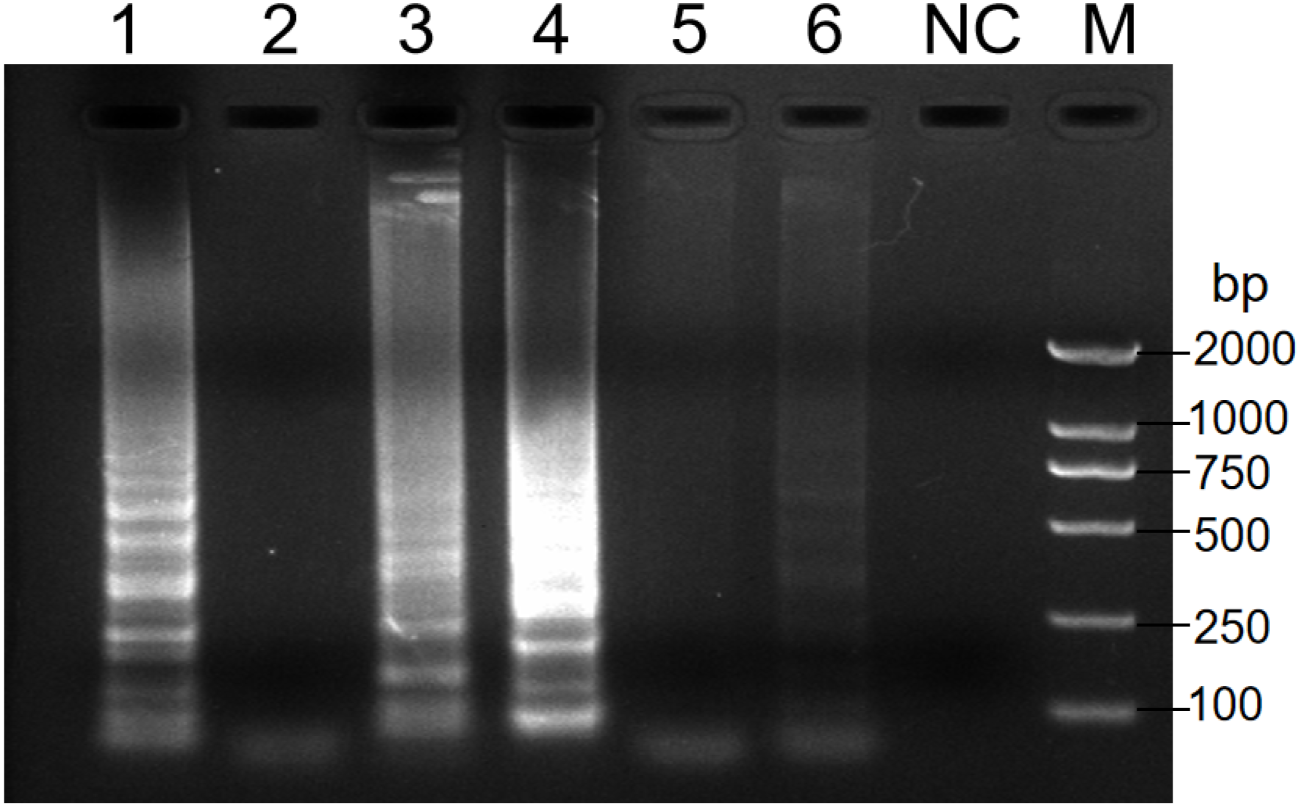
Analysis of the products after amplification with the indicated primers. Electrophoresis was performed on a 1.5% agarose gel to analyze the amplification products of RT-PSR assays with six different sets of primers. Lanes 1-6 indicate primers no. 1 to 6 respectively. Lane NC, negative control (RNase-Free ddH_2_O); Lane M, DL 2000 DNA marker.

Analysis of the RT-PSR results revealed that when primer sets 1, 3 and 4 were used, a distinct DNA ladder was visible on the agarose gel (**Figure 1**). This clearly indicated successful nucleic acid amplification under isothermal conditions. Conversely, no amplification was observed with the remaining three primer sets. This could potentially be due to sub-optimal amplification parameters. Among all primer sets, primer set 1 exhibited the highest amplification efficiency. As a result, it was selected for subsequent RT-PSR experiments as detailed in **Table 1**.

### 3.2 RT-PSR Optimization

**Figure 2:**
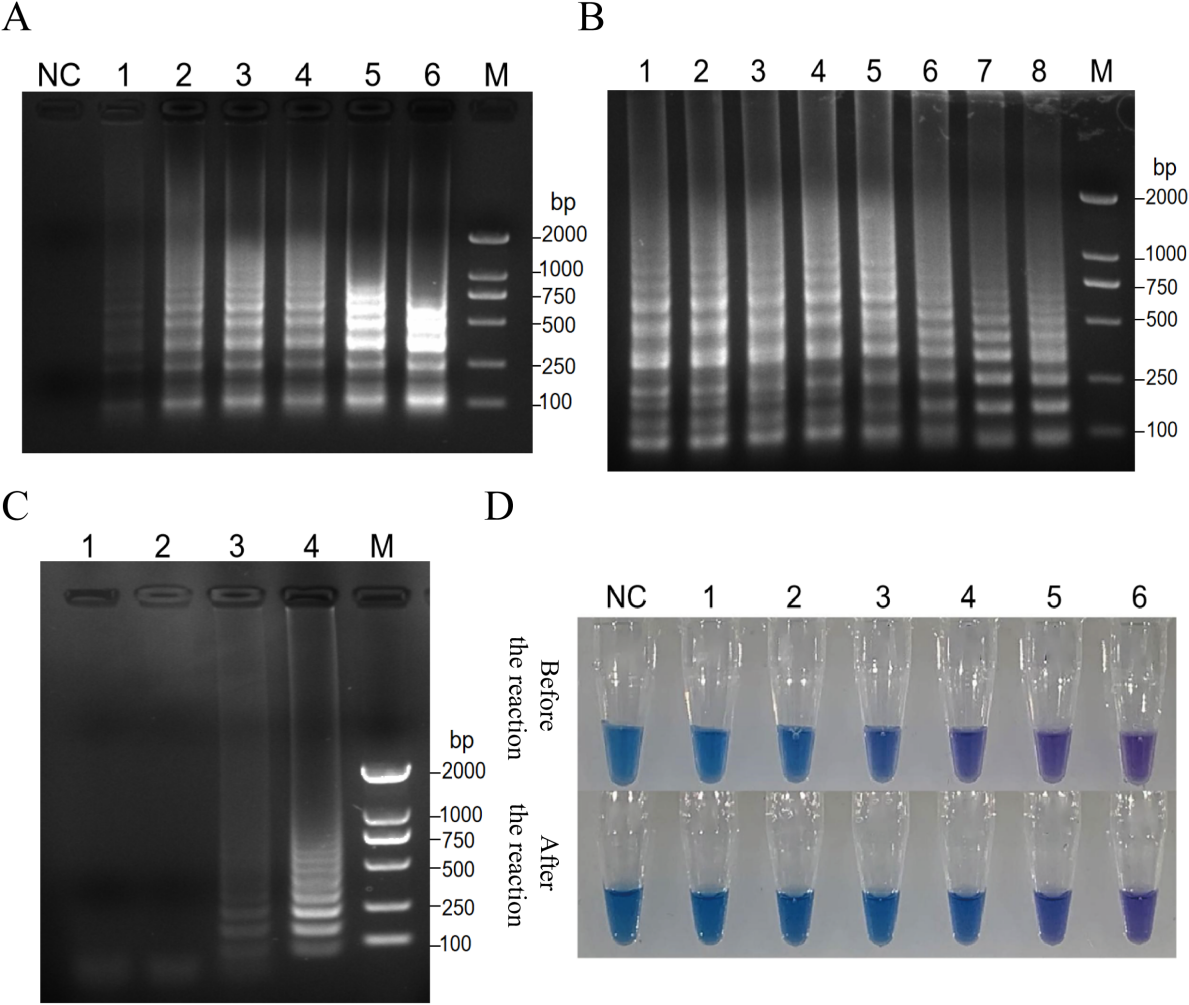
Key reaction parameters for the RT-PSR assay, including primer concentration, reaction temperature, and duration, were optimized using CVA6 RNA. **(A)** Analysis of the products after amplification with primer 1 at the indicated primer concentrations. Electrophoresis was performed on a 1.5% agarose gel to analyze the amplification products of RT-PSR assays with primer 1 at the primer concentrations of 1.6 µmol/L (Lane 1); 2.0 µmol/L (Lane 2), 2.4 µmol/L (Lane 3), 2.8 µmol/L (Lane 4), 3.2 µmol/L (Lane 5), and 3.6 µmol/L (Lane 6), respectively. Lane NC, negative control (RNase-Free ddH_2_O); Lane M, DL 2000 DNA marker. **(B)** Analysis of the product after amplification with primer 1 at the indicated temperatures. RT-PSR amplification was carried out at the temperatures of 59°C (Lane 1), 59.6°C (Lane 2), 60.8°C (Lane 3), 62.4°C (Lane 4), 64.5°C (Lane 5), 66.2°C (Lane 6), 67.3°C (Lane 7), and 68.0°C (Lane 8), respectively. Lane M, DL 2000 DNA marker. **(C)** Analysis of the product after amplification with primer 1 at 64.5°C for the indicated time periods. RT-PSR amplification was performed for 30 min (Lane 1), 40 min (Lane 2), 50 min (Lane 3), and 60 min (Lane 4), respectively. Lane M, DL 2000 DNA marker. **(D)** The optimization of Mg^2+^ concentrations in the reaction. Visual detection of RT-PSR amplification products produced at different concentrations of Mg^2+^. NC, negative control (DEPC-treated water); Tube 1, 2.0 mmol/L; Tube 2, 4.0 mmol/L; Tube 3, 6.0 mmol/L; Tube 4, 8.0 mmol/L; Tube 5, 10 mmol/L; Tube 6, 12 mmol/L.

Key reaction parameters for the RT-PSR assay, including primer concentration, reaction temperature, and duration, were optimized using CVA6 RNA. The results of the optimization for primer concentration show that at a final concentration of 1.6 µmol/L, the bands were faint. However, as the concentration increased above 2.0 µmol/L, the amplification bands became stronger, with 2.4 µmol/L identified as the optimal concentration (**Figure 2A**). Temperature optimization showed that the range of 59-68°C produced minimal variability in band intensity (**Figure 2B**). A reaction temperature of 65°C was determined to be optimal. Time optimization revealed that no bands formed at 65°C until after 40 minutes, with the bands reaching their best intensity after 60 minutes (**Figure 2C**). The final optimized conditions for CVA6 detection were 65°C for 60 minutes, with a Mg^2+^ concentration of 8 mmol/L and 25 µL reaction volumes containing HNB dye for improved differentiation between positive and negative results (**Figure 2D**).

### 3.3 Sensitivity and specificity of the RT-PSR Assay

**Figure 3:**
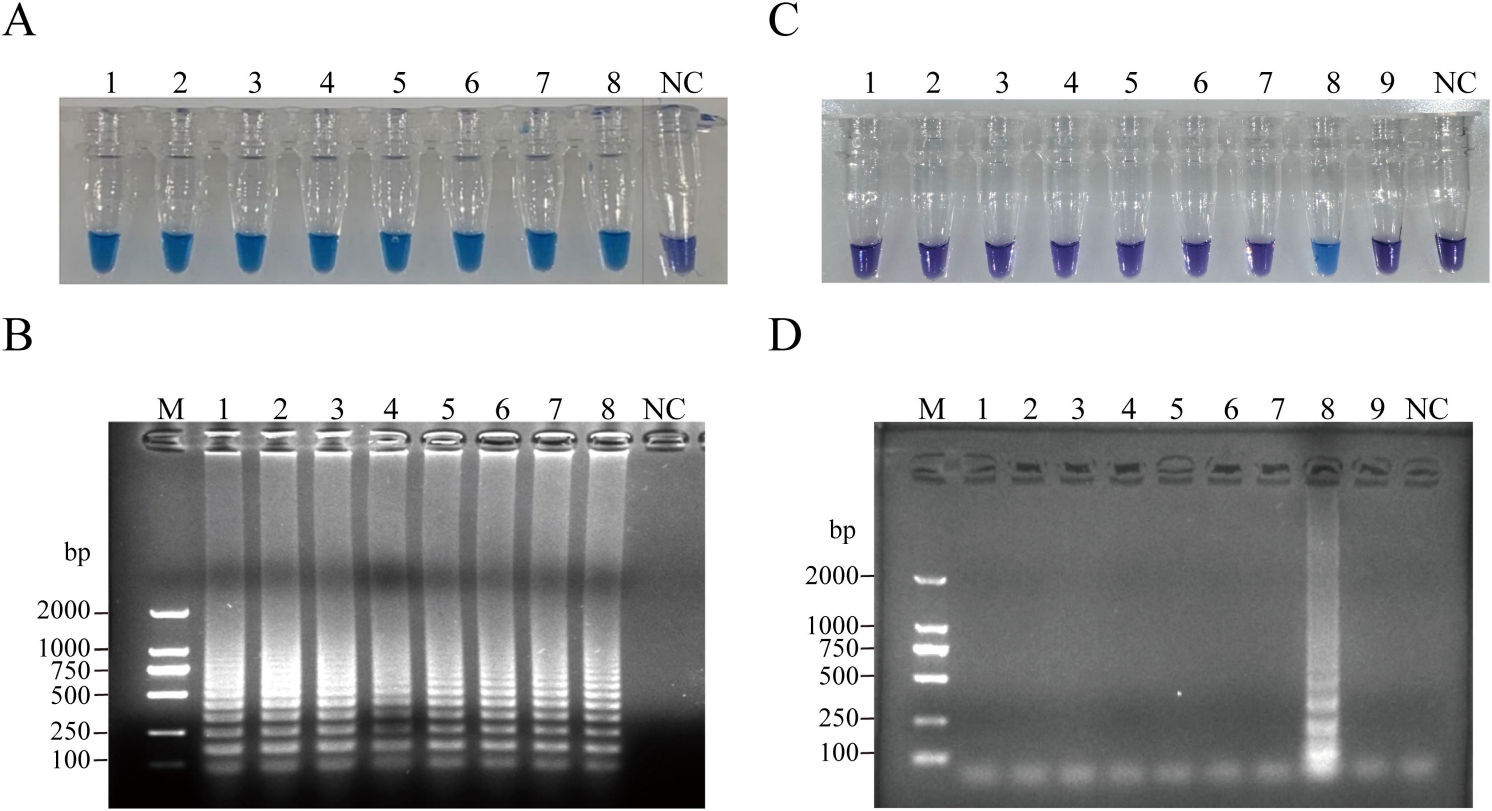
Sensitivity of the RT-PSR amplification for CVA6. **(A)** Visual detection of RT-PSR amplification products utilizing the HNB indicator. Tube 1, 1 × 10^8^ copies/µL; Tube 2, 1 × 10^7^ copies/µL; Tube 3, 1 × 10^6^ copies/µL; Tube 4, 1 × 10^5^ copies/µL; Tube 5, 1 × 10^4^ copies/µL; Tube 6, 1 × 10^3^ copies/µL; Tube 7, 1 × 10^2^ copies/µL; Tube 8, 1 × 10^1^ copies/µL; NC, negative control (DEPC-treated water). **(B)** Agarose gel electrophoresis of RT-PSR amplification products. Lane M: DL 1,000 DNA marker. Lane 1, 1 × 10^8^ copies/µL; Lane 2, 1 × 10^7^ copies/µL; Lane 3, 1 × 10^6^ copies/µL; Lane 4, 1 × 10^5^ copies/µL; Lane 5, 1 × 10^4^ copies/µL; Lane 6, 1 × 10^3^ copies/µL; Lane 7, 1 × 10^2^ copies/µL; Lane 8, 1 × 10^1^ copies/µL; NC, negative control (RNase-Free ddH_2_O). **(C)** Visual detection of RT-PSR amplification products. Tube 1, hepatitis B virus; Tube 2, hepatitis C virus; Tube 3, respiratory syncytial virus; Tube 4, cytomegalovirus; Tube 5, EV71; Tube 6, CVA10; Tube 7, CVA16; Tube 8, CVA6; Tube 9, rotavirus; NC, negative control (RNase-Free ddH_2_O). **(D)** Agarose gel electrophoretic profile of RT-PSR amplification products from 9 viral strains. Lane M: DL 2,000 DNA marker. Lane 1, hepatitis B virus; Lane 2, hepatitis C virus; Lane 3, respiratory syncytial virus; Lane 4, cytomegalovirus; Lane 5, EV71; Lane 6, CVA10; Lane 7, CVA16; Lane 8, CVA6; Lane 9, rotavirus; Lane NC, negative control (RNase-Free ddH_2_O).

The analytical sensitivity of RT-PSR was assessed by performing a 10-fold serial dilution of plasmid templates. The limit of detection for RT-PSR was determined to be 10 copies/µL. When the target template concentration exceeded or equaled 1 × 10^1^ copies/µL, the reaction solution exhibited a sky-blue color under natural light, while RNase-Free ddH_2_O resulted in a violet coloration of the reaction mixture. These findings were further confirmed by agarose gel electrophoresis (**Figures 3A, B**). The specificity of the assay was evaluated using a panel of 8 strains. Only CVA6 produced a sky-blue color, while all other strains yielded violet results (**Figure 3C**). The electrophoretic results clearly showed that amplicons were generated solely in CVA6 samples (**Figure 3D**). No non - specific amplification occurred in any of the control virus samples, effectively highlighting the high specificity of the assay.

### 3.4 Clinical specimens testing

**Figure 4:**
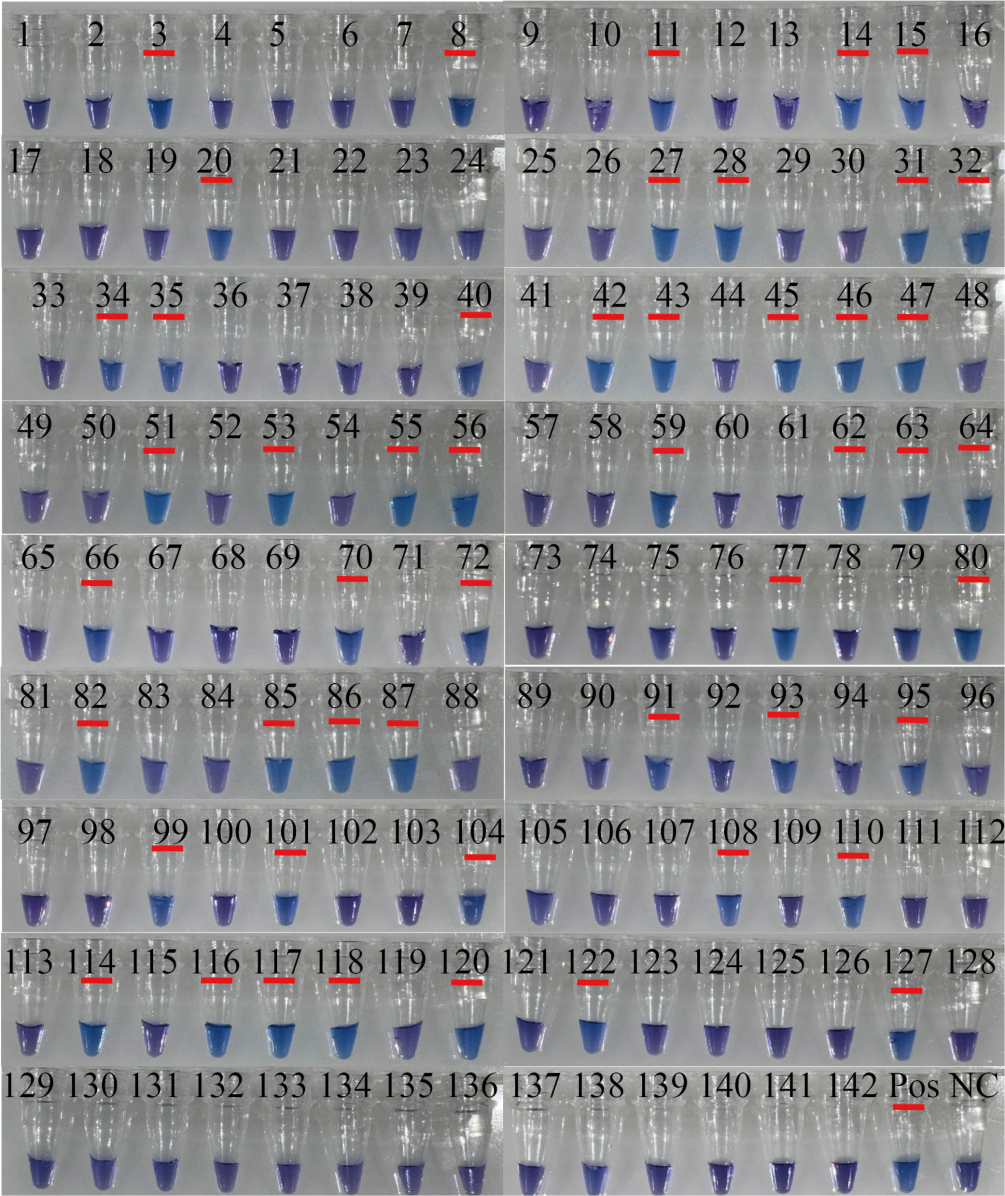
The results of visualized RT-PSR to detect clinical samples. Visual detection of RT-PSR amplification products from clinical samples (tube no. 1 to 142). NC, negative control (RNase-FreeH_2_O); Pos, 10^8^ copies/µL the standard plasmid.

RT-PSR and TaqMan real-time RT-PCR were used to test 142 clinical samples. Both methods yielded 50/142 positive results (**Figure 4**). Using TaqMan real-time RT-PCR as a reference, the RT-PSR assay demonstrated a 100% positive and negative agreement, with a total agreement of 100%, a Youden index of 1, and a Kappa coefficient of 1 (**Table 2**). This indicates that the RT-PSR method is highly consistent with qRT-PCR results.

**Table 2:** The RT-PSR assay was comparatively evaluated with the commercial CVA6 kits for the detection of CVA6 in throat swab samples from human patients.

RT-PSR	RT-PCR		Total
	Positive	Negative	
Positive	50	0	50
Negative	0	92	92
Total	50	92	142

## 4 Discussion

This study successfully developed a highly sensitive and specific RT - PSR assay for rapid visual detection of CVA6, a primary causative agent of HFMD and HA. By targeting the VP1 gene and employing HNB as a visual indicator, this assay is capable of detecting as few as 10 copies/μL within 60 minutes at 65°C. When evaluated using 142 clinical throat swab samples, the RT-PSR method demonstrated complete concordance with quantitative RT-PCR (qRT-PCR), validating its efficacy for on-site clinical diagnostics and highlighting its potential utility in resource-constrained environments.

In recent years, the extensive administration of EV71 vaccines has notably reduced the incidence of EV71-associated HFMD cases. Research has revealed that CVA6 has become the primary pathogen in severe HFMD cases across diverse regions in China (Lu et al., 2024b). The growing prevalence of CVA6 in recent years has contributed to an elevated incidence of HFMD among children (Noisumdaeng and Puthavathana, 2023). However, the clinical manifestations of CVA6-related HFMD differ from those of other types. Besides typical symptoms like fever, sore throat, and herpes simplex on the skin of the hands, feet, and mouth, atypical features such as herpetic lesions and nail abnormalities are more frequently observed (Horsten et al., 2018). This situation underscores the insufficiency of infection control strategies that solely target typically symptomatic patients in preventing viral transmission, which may lead to misdiagnoses (Chen et al., 2023). Thus, performing CVA6 enterovirus detection in patients with atypical rashes is of great clinical significance for improving disease diagnosis and identification. In this study, we have developed a novel isothermal nucleic acid amplification method by designing specific primers targeting the conserved sequence of VP1 gene in CVA6. The established method exhibited excellent performance in terms of sensitivity and specificity. In addition, this method is well-suited for POCT (point-of-care testing) and can save approximately 1 hour in testing time compared to qRT-PCR.

The primers were designed on the basis of VP1 sequences. The VP1 capsid protein gene of enterovirus is genetically correlated with the serotype of enterovirus (Liu et al., 2024). In this study, these designed primers demonstrated remarkable specificity for the CVA6 genotype. No cross-reactivity was detected with CVA16, EV71, and CVA10 genotypes. Compared to LAMP, recombinase polymerase amplification, and helicase-dependent amplification, RT-PSR presents several advantages. RT-PSR requires fewer primers than LAMP, streamlining primer design and cutting costs. Similar to LAMP and HDA, it functions at a single temperature. However, it attains high sensitivity and specificity comparable to RPA without the need for recombinase or helicase proteins, which can complicate reaction setup and increase costs. Additionally, RT-PSR adopts a visual detection method with HNB, enabling straightforward result interpretation without complex equipment. This makes it especially suitable for point-of-care testing and resource-limited settings (Dixit et al., 2024).

Several indicators have been utilized to report the amplification process. One approach is to use a pH indicator such as phenol red and conduct the reaction in a weakly buffered environment. As the chain reaction progresses, the pH decreases, causing a visible color change from red to yellow. However, it should be noted that samples do not alter the PH of the reaction (Dao Thi et al., 2020). SYBR green is another common indicator. Positive reactions fluoresce brightly green, while negative samples maintain an orange color. Nevertheless, as more and more double-stranded DNA chimeras are formed due to continuous DNA amplification product generation in the reaction, SYBR Green inhibits the strand displacement activity of Bst DNA polymerase, thus impeding the reaction rate (Xu et al., 2019). In this study, we selected HNB because it has minimal interference. HNB is a metal ion - binding indicator dye that binds to Mg^2+^ ions and changes color according to the Mg^2+^ concentration in the reaction mixture. When the Mg^2+^ concentration is 6 mM or higher, HNB appears purple; below 6 mM, it turns sky blue (Pauly et al., 2023).

The assay demonstrated a high sensitivity in CVA6 detection, with a minimum limit of detection (LOD) of 1 × 10^1^ copies/µL, which is approximately equivalent to the sensitivity of conventional RT-PCR of 0.9 × 10^1^ copies/µl. RT-PSR is an isothermal amplification method. Although it may sacrifice some sensitivity compared to traditional methods, it offers substantial advantages in terms of convenience, specificity, and speed. In 2020, He et al (He et al., 2020). established an RT-PSR for CVA16, achieving a sensitivity range of 2.4 × 10^2^ and 2.4 × 10^1^ copies/µL. Combined with our established method will greatly increase the diagnostic rate of HFMD. Only three previous studies of PSR estimated diagnostic indicators such as sensitivity, specificity and LOD. The reported specificities of PSR were 92%, 93.7% and 100% respectively (Sharma et al., 2020; Milton et al., 2021; Tomar et al., 2020). Our study showed higher specificity, despite having slightly lower sensitivity compared to qRT-PCR-based assays. This closed-tube real-time detection method allows for easy visual result interpretation, making it more convenient and appropriate for primary hospitals and on-site detection in resource-limited regions.

Overall, this study successfully developed a visual RT-PSR assay for CVA6. The assay exhibits remarkable sensitivity, rapid reaction times, and high specificity. In addition, it enables non-invasive analysis and is user-friendly. Despite these achievements, the study has several limitations. The performance of the RT-PSR assay is highly reliant on the optimized concentration of Mg²⁺ and reaction temperature. Therefore, standardized conditions and strict operational procedures are essential. Currently, the assay has only been validated using throat swab samples. Thus, further research is required to explore its applicability to other sample types. Another shortcoming is the limited number of clinical samples included in this study. As a result, a comprehensive evaluation of the assay’s performance relative to other molecular diagnostic techniques in a wider range of clinical settings is still needed. For future research, a significantly larger and more diverse sample population should be incorporated. Also, under various field conditions, systematic comparisons between the RT - PSR detection method and established diagnostic techniques should be carried out. These steps will help to better assess the reliability and generalizability of the RT - PSR assay. Notwithstanding these limitations, this innovative RT - PSR assay offers practical technical support for real - time, on - site testing. It holds particular promise for resource - constrained rural and suburban areas. As such, it can contribute to the expansion of hand, foot, and mouth disease (HFMD) control measures, as well as management strategies for other viral diseases.

## Data Availability

The original contributions presented in the study are included in the article/supplementary material. Further inquiries can be directed to the corresponding authors.
